# Minimal-active-space multistate density functional theory for excitation energy involving local and charge transfer states

**DOI:** 10.1038/s41524-021-00624-3

**Published:** 2021-09-17

**Authors:** Ruoqi Zhao, Christian P. Hettich, Xin Chen, Jiali Gao

**Affiliations:** 1 Institute of Systems and Physical Biology, Shenzhen Bay Laboratory, Shenzhen 518055, China.; 2 Institute of Theoretical Chemistry, Jilin University, Changchun, Jilin Province 130023, China.; 3 Department of Chemistry and Supercomputing Institute, University of Minnesota, Minneapolis, Minnesota 55455, USA.; 4 Beijing University Shenzhen Graduate School, Shenzhen 518055, China.

## Abstract

Multistate density functional theory (MSDFT) employing a minimum active space (MAS) is presented to determine charge transfer (CT) and local excited states of bimolecular complexes. MSDFT is a hybrid wave function theory (WFT) and density functional theory, in which dynamic correlation is first incorporated in individual determinant configurations using a Kohn–Sham exchange-correlation functional. Then, nonorthogonal configuration-state interaction is performed to treat static correlation. Because molecular orbitals are optimized separately for each determinant by including Kohn–Sham dynamic correlation, a minimal number of configurations in the active space, essential to representing low-lying excited and CT states of interest, is sufficient to yield the adiabatic states. We found that the present MAS-MSDFT method provides a good description of covalent and CT excited states in comparison with experiments and high-level computational results. Because of the simplicity and interpretive capability through diabatic configuration weights, the method may be useful in dynamic simulations of CT and nonadiabatic processes.

## INTRODUCTION

Charge transfer (CT) states are electronically excited states which are of fundamental importance in materials and chemical processes, including those in photosynthesis, photoreceptor proteins, catalysis, and photovoltaic devices. Two questions are intertwined and need to be considered together:^1^ (1) an accurate description of the electronic excitation of CT states and (2) an efficient and chemically intuitive representation of the potential energy surfaces for the subsequent nuclear dynamic simulations. In principle, high-level quantum-chemical methods can be used; however, in practice, it is often difficult to find an approach that can satisfy both requirements simultaneously. Furthermore, most computational models for excited states are based on multi-configuration self-consistent-field (MCSCF) approaches such as the complete-active-space self-consistent-field (CASSCF) method. Even at the simplest level of single-excitation configuration interaction (CIS) and linear-response time-dependent density functional theory (TDDFT), which is known to perform poorly on CT states, the large number of configurations included in these calculations makes the interpretation of CT states difficult^2–5^. CT states are long-ranged and highly sensitive to donor-acceptor distance, typically having intersections with all local states in the form of both conical intersections and avoided curve crossings. Thus, the assignment of CT states can be difficult as shown in a recent study of bimolecular complexes that include inter-fragment CT interactions^2,6,7^. In this article, we present an approach that employs a minimal active space (MAS) in multistate density functional theory (MSDFT) to treat CT states as well as local valence excitations on a range of bimolecular complexes. Importantly, the present method provides a straightforward interpretation of results.

For large chromophores of materials interest, TDDFT is a desired approach to model excited states and charge separation processes^3,8–10^, and a number of developments have made these methods more reliable for treating CT states^11,12^, including double hybrid functionals^13,14^ and range-separated techniques^15,16^ along with the optimization of the separation parameter specifically for a given system^17,18^. Recent studies showed that restricted open-shell Kohn–Sham DFT and exciton models may present other ways of modeling CT states^19–22^. Nevertheless, the performance of TDDFT on CT states is often system and functional-dependent^3,8,10,12,16,23^, and it is not clear if a double hybrid functional is viable for treating large chromophores in photo-systems^14^. It was pointed out that the uncertainties in TDDFT calculations demand benchmark tests to calibrate economic approaches for CT states^2^. To this end, a set of two-component molecular complexes that include local valence excitations and CT states has been investigated at the EOM-CCSDT (equation of motion coupled cluster with singles, doubles, and triples) and EOM-CCSDT-3 (iterative EOM-CCSDT) levels of theory^2^. Subsequently, the performance of a family of lower-cost CC models was evaluated, including EOM-CCSD (equation of motion coupled cluster with singles and doubles), CC2 (second-order approximations to CCSD), and ADC(2) (second-order algebraic diagrammatic construction) along with the third-order CC3 and non-iterative EOM-CCSD(T)(a)* alternatives^2,6^. The test was further extended to the spin-component-scaled (SCS) CC2 and ADC(2), showing significant reduction in error over the original CC2 and ADC(2) calculations^7^.

MSDFT was developed for treating CT reactions such as proton-coupled electron transfer^24^ and singlet fission of pentacene monolayer materials^25^, in which a balanced treatment of dynamic and static correlation is important^26–29^. Our aim is to use a small set of localized configurations with well-defined physical properties, called diabatic states such as those in Marcus theory for electron transfer, to represent CT and excited states of molecular complexes^30,31^. Consequently, charge-localized and locally excited diabatic states can be defined to form an active space of basis configurations^32,33^. In addition, the method can be used as an interpretive tool in energy decomposition analysis to quantify CT energy and exciton coupling^34,35^. MSDFT is a hybrid wave function theory (WFT) and DFT, in which dynamic correlation is modeled through a Kohn–Sham density functional at the level of each individual determinant^26,27^. As a result, dynamic correlation is incorporated in each determinant configuration, and the energies of the adiabatic states are obtained by a “dynamic-then-static”^36–38^ type of nonorthogonal state interaction (NOSI)^39–43^. Coupled with separate optimizations of the orbitals in all determinant configurations, we anticipate that only a minimal number of basic configurations, essential to the problem of interest, is needed in MSDFT to treat low-lying valence and CT excited states. This expectation is confirmed in the present investigation.

For comparison, there has been much recent progress in the development of ensemble DFT (EDFT) based on a variational principle of statistical ensembles of ground and excited states^44–48^, where the excitation energies can be extracted. Although the use of an exact exchange approximation brings hope to model CT excitations^49^, practical applications have so far been scarce^50^. EDFT treats a collection of adiabatic states within the realm of KS-DFT without consideration of state interactions. On the other hand, adiabatic ground and excited states are obtained in MSDFT by NOSI in which dynamic contributions are introduced into electronic coupling^27^. To this end, a spin-restricted EDFT has been described, effectively introducing coupling by optimizing fractional occupations^51,52^, and a DFT/MRCI method has been reported, which employs empirically scaled two-electron integrals^53^. There have also been a number of multiconfigurational DFT (MC-DFT) models in the literature^54–59^. Among the early works of this category are those by Savin and coworkers, who used the on-top pair density to treat multi-determinant states^60^ and introduced a local measure of density aimed at removing the fraction of correlation energy from the DFT functional that has already been expressed in the MCSCF wave function^61^. The latter approach has been extended to open-shell systems and multi-reference perturbation theory^54–56^. In this regard, MC-DFT also follows the KS approximation and, in practice, makes use of KS functionals with transformed densities. Common to all these methods is the use of a common set of orbitals for all states. In contrast, in MSDFT, the orbitals of each determinant state are separately optimized.

## RESULTS AND DISCUSSION

### Theoretical method

The energy density functional of MSDFT for the adiabatic ground and excited states is given by^26,27^

(1) $$E_{I}^{\text{MS}}\left[\rho_{I}\right]=\sum_{A}C_{AI}^{2}H_{A}\left[\rho_{A}\right]+2\sum_{A>B}C_{AI}C_{BI}H_{AB}^{\text{TDF}}\left[\rho_{AB},\Psi_{A},\Psi_{B}\right]$$

where $E_{I}^{\text{MS}}\left[\rho_{I}\right]$ is the energy of adiabatic state *I*, *H_A_* [*ρ_A_*] is the Kohn–Sham DFT energy for block-localized determinant *A* in the active space, corresponding to a diagonal matrix element of the NOSI Hamiltonian, $H_{AB}^{\text{TDF}}\left[\rho_{AB},\Psi_{A},\Psi_{B}\right]$ is a transition density functional (TDF) of the off-diagonal matrix element, defining electronic coupling between states *A* and *B*, and the coefficients {*c*} are obtained by solving the generalized secular equation HC = SCE. Formally, the determinant state Ψ*_A_* is strictly defined by a constrained KS-DFT^62^ whose constraining potential forces the KS-state to reproduce the energy of a contracted state of the full configuration interaction (FCI) wave function through singular value decomposition (SVD)^27^. In the present study, *H_A_*[*ρ_A_*] is approximated using block-local excited KS-DFT^26^, with the slight complication that nonorthogonal orbitals are employed.

The TDF of the off-diagonal matrix elements of the NOSI Hamiltonian H is new (absent in KS-DFT). Specifically, TDF can be separated into two terms: a wave function contribution between nonorthogonal KS-determinants Ψ*_A_* and Ψ*_B_*^41,42^, and a TDF correlation energy $E_{AB}^{\text{TDF}}$^26,27,32,33,63^,

(2) $$H_{AB}^{\text{TDF}}=\langle \Psi_{A}^{\text{BLE}}\left|H\right|\Psi_{B}^{\text{BLE}}\rangle +E_{AB}^{\text{TDF}}\left[\rho_{AB},\Psi_{A}^{\text{BLE}},\Psi_{B}^{\text{BLE}}\right]$$

where *H* is the electronic Hamiltonian, and *ρ_AB_* is the transition density of determinants Ψ*_A_* and Ψ*_B_*. $E_{AB}^{\text{TDF}}$ has the formal definition as the difference between the electronic coupling of two SVD-contracted FCI states, denoted by $H_{AB}^{\text{FCI}-\text{SVD}}$, and that of two constrained KS-determinants $\left(\langle \Psi_{A}^{\text{BLE}}\left|H\right|\Psi_{B}^{\text{BLE}}\rangle \right)$^27^. Therefore, the MSDFT eigenstates in Eq. 1 would be exact in the sense that they would reproduce the FCI energies, had the exact constraining potential been known for each determinant.

In practical applications, the exact functional form of $E_{AB}^{\text{TDF}}$, just as the exchange-correlation functional in Kohn–Sham DFT, is unknown and an explicit approximate functional has not yet been developed. However, under special situations such as the present CT and local excitations involving spin coupling interactions, its value can be consistently determined with the Kohn–Sham exchange-correlation functional used to optimize the block-localized (a form of constrained) Kohn–Sham determinants Ψ*_A_* and Ψ*_B_*, avoiding or minimizing any double counting of electron correlation^28,64,65^. In particular, for spin-pairing interactions between two unpaired electrons, the TDF energy can be obtained by enforcing the degeneracy condition of spin components of the triplet states as proposed by Ziegler et al.^65,66^,

(3) $$E_{AB}^{\text{TDF}}\left[\rho_{AB},\Psi_{↑↓},\Psi_{↓↑}\right]=E_{C}^{\text{KS}}\left[\rho \left(\Psi_{↑↑}\right)\right]-E_{C}^{\text{KS}}\left[\rho \left(\Psi_{↓↑}\right)\right]$$

where $\Psi_{↑↓}$ and $\Psi_{↓↑}$ are the determinants (*A* and *B*) of different spin combinations of two unpaired electrons, $E_{C}^{\text{KS}}\left[\rho \left(\Psi_{↑↑}\right)\right]$ and $E_{C}^{\text{KS}}\left[\rho \left(\Psi_{↓↑}\right)\right]$ are, respectively, the correlation energies of KS-DFT using the determinant for the high-spin (both spins up) determinant $\Psi_{↑↑}$ and a mixed-spin determinant $\Psi_{↓↑}$ (equally $\Psi_{↑↓}$)^28^. This approach has been generalized to any spin coupling cases^65^. An example using a triplet reference for singlet-triplet splitting may be found in the spin-restricted density functional theory for biradicals^51,67^. For other situations in this work, we used the overlap-scaled correlation energy to approximate the TDF energy for electronic coupling:

(4) $$E_{AB}^{\text{TDF}}=\frac{1}{2}S_{AB}^{\text{KS}}\left[E_{C}^{\text{KS}}\left(\rho_{A}\right)+E_{C}^{\text{KS}}\left(\rho_{B}\right)\right]$$

where $S_{AB}^{\text{KS}}=\langle \Psi_{A}\left|\Psi_{B}\right.\rangle$, the overlap between two Kohn–Sham determinants ^26,27^. Throughout this study, we employed the Minnesota M06–2X functional^68^, denoted as MSDFT@M06–2X, and cc-pVDZ basis sets, which was used to match that in the work of Kozma et al. for comparison^2,7^.

### CT resonance states

Recently, we investigated the significance of CT stabilization of valence excited states on the naphthalene excimer complex using MSDFT (Supplementary Fig. 1)^30^ and introduced a block-localized excitation (BLE) technique to form the MAS. Here, we extend that study to the excimer complex of anthracene molecules and examine the effect of orbital overlap on CT resonance coupling by sliding a monomer along the direction of the long molecular axis (*x*-axis). First, there are two characteristic $\pi \to \pi^{*}$ singlet excited states of linear polyacenes, corresponding to the *L_a_* state (Platt notation or ^1^B_2u_)^69–71^, predominantly of HOMO → LUMO character with a transition density from the ground state that is polarized along the short *y*-axis, and the *L_b_* (^1^B_3u_) states, which is a mixture of HOMO → LUMO + 1 and HOMO–1→LUMO transitions. For benzene and naphthalene, the *L_b_* state is lower than *L_a_*, but the order is reversed for anthracene and larger polyacenes^70,72^. Similar to the naphthalene excimer^30^, we include three (3) locally excited configurational state functions (CSF) for each monomer, corresponding to HOMO → LUMO, HOMO → LUMO + 1, and HOMO–1→LUMO excitations, and a pair of inter-fragment CT states to describe the low-energy states^73^. Thus, the present MAS in MSDFT calculations consists of eight spin-adapted CSFs plus the Kohn–Sham ground state. The monomer geometry in the ground state, optimized using B3LYP/aug-cc-pVTZ, is used and the monomers are placed at the face-to-face configuration at a separation of 3.2 Å, close to the minimum distance of the first singlet excimer complex^74^. Since *L_b_* is little affected by the CT states^30^, we focus on the singlet *L_a_* and triplet states in the discussion of anthracene excimer.

**Fig. 1:**
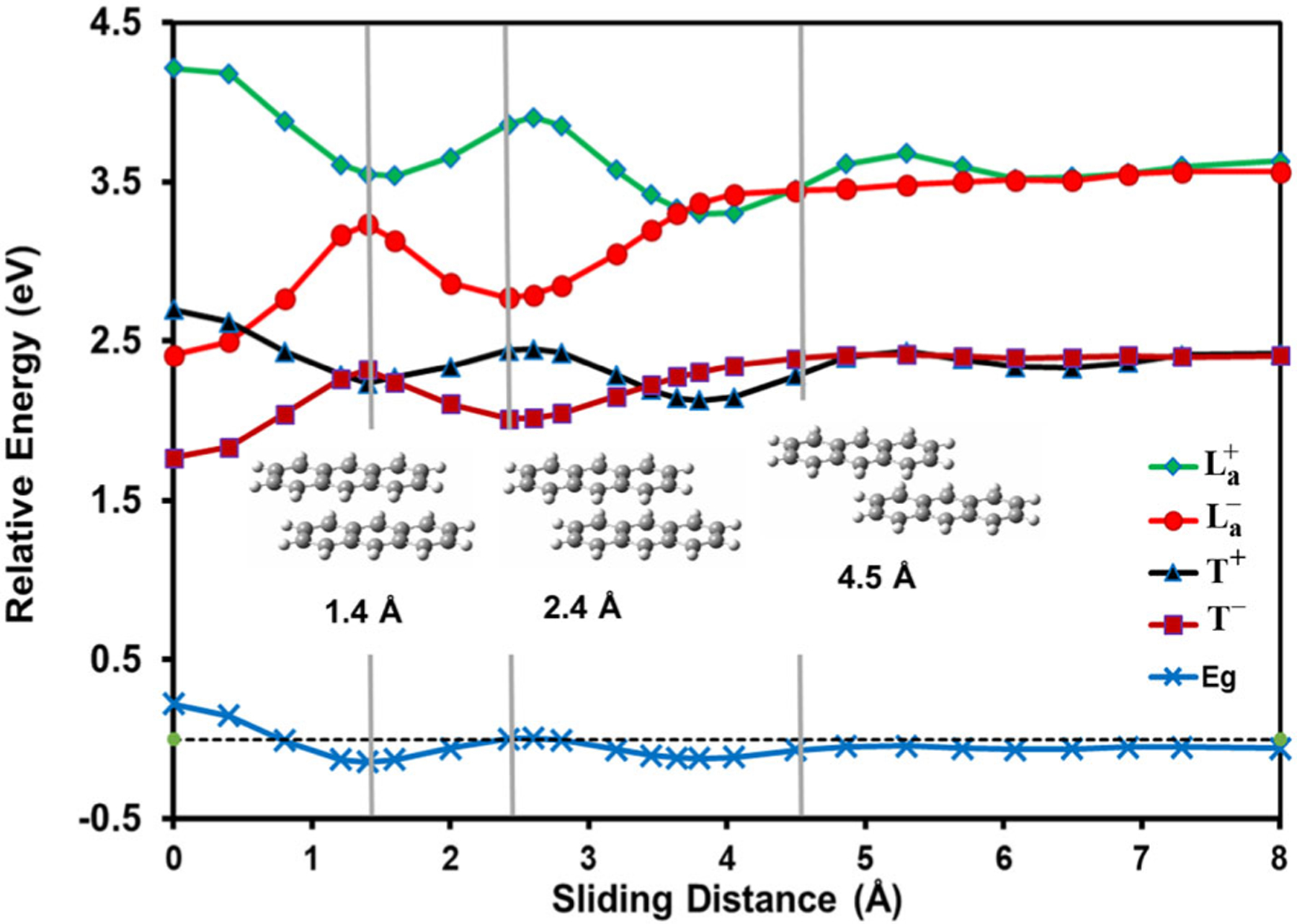
Computed potential energies curves for the anthracene excimer. Shown are the adiabatic ground state (Eg), the in-phase and out-phase combinations of locally excited singlet (L_a_^+^ and L_a_^−^) and triplet (T^+^ and T^−^) states, stabilized by mixing with the resonance states of forward and backward charge transfer states.Vertical gray lines indicate the position of the shown structures on the abscissa. Energies are given in electron volts relative to the separated monomers in the ground state.

Depicted in Fig. 1 are the potential energy curves along the *x*-axis for the singlet ($L_{a}^{-}$ and $L_{a}^{+}$) and triplet (*T*^−^ and *T*^+^) states of anthracene dimer, corresponding to the out-phase and in-phase combinations of the monomer singlet *L_a_* and triplet states. First, the computed vertical excitation energies of anthracene are 3.66 eV for the singlet *L_a_* state and 2.47 eV for the triplet state. A recent quantum Monte Carlo calculation yielded vertical excitation energy of 3.64 eV for the *L_a_* state^75^. The computed adiabatic transitions are 3.40 and 2.06 eV, respectively, which may be compared with the corresponding experimental values of 3.43 and 1.85 eV^76,77^. Clearly, the adiabatic triplet state is sensitive to geometry relaxation, suggesting that the energy curves of the triplet states may be overestimated by about 0.4 eV in Fig. 1^76,77^.

The minimum-energy arrangement of both the singlet and triplet state of the anthracene excimer is in the face-to-face configuration (Fig. 1). The two local (monomer) ^1^B_2u_(*L_a_*) states and two triplet states respectively interact to yield a pair of in-phase ($L_{a}^{+}$ and *T*^+^) and of out-phase ($L_{a}^{-}$ and *T*^−^) exciton combinations. Importantly, the forward and backward CT resonance states are strongly mixed with the valence exciton states, leading to a binding energy of −1.25 eV for the $L_{a}^{-}$ state and of −0.58 eV for the *T*^−^ state. Without including CT stabilization, the exciton coupling of two local ^1^B_2u_ states only produces resonance energy of just −0.38 eV (30%) in the singlet state and the coupling between two local triplet states results in a net repulsive interaction by 0.08 eV, consistent with the findings in the naphthalene excimer^30^. For comparison, binding energies of 1.72 and 1.09 eV, respectively, for the singlet and triplet excimers have been reported from a scaled-opposite-spin (SOS) CIS(D_0_) calculation^74^. The importance of CT states has been attributed to account for the ultrafast conversion of singlet fission in pentacene monolayer materials^25^.

Figure 1 reveals that the anthracene excimer has a singlet-triplet energy gap of 0.65 eV between the $L_{a}^{-}$ and *T*^−^ states, which is in good accord with a computational value of 0.63 eV using the SOS-CIS(D_0_) method^74^. The singlet-triplet energy gap is significantly reduced in the excimer complex relative to that of the monomer (1.19 eV) because the CT resonance states strongly enhance exciton coupling of local excitations, stabilizing the $L_{a}^{-}$ state and giving rise to a large energy separation between $L_{a}^{-}$ and $L_{a}^{+}$. On the other hand, CT stabilization of the triplet state is smaller than that of the *L_a_* state, leading to a differential singlet-triplet energy gap. The effect of orbital phase-matching on the singlet-triplet gap is illustrated in Fig. 1 as the two monomers are slid along the *x*-axis. At a sliding distance of 1.4 Å relative to the face-to-face configuration (Fig. 1), the overlap between the two locally excited states reaches a minimum, at which point the exciton coupling has weak interactions, reducing $L_{a}^{-}-L_{a}^{+}$ splitting to only 0.32 eV. This represents a net reduction by 1.48 eV relative to the exciton coupling (1.80 eV) in the face-to-face configuration. The origin of the large variation of exciton coupling along the molecular long axis can be attributed to reduced CT mixing with the local valence states from the analysis of structure weight. It is interesting to note that the singlet-triplet gap increases at this distance as a result of the opposite trend between $L_{a}^{-}$ and *T*^+^ (Fig.1). Three periods of oscillations are clearly seen in the $L_{a}^{-}$ and $L_{a}^{+}$ coupling, corresponding to maximum overlaps of three, two, and one aromatic rings. Exciton coupling diminishes beyond about 6 Å, about half-ring left in dimer overlap, which is in good accord with a study of substituted anthracene excimers using TDDFT^78^. Note that although the *T*^−^ state oscillates synchronously with the $L_{a}^{-}$ state, the variation of the *T*^+^ state follows reciprocally, leading to a curve crossing with $L_{a}^{-}$ at about 0.9 Å.

An understanding of electronic coupling in terms of phase-matching of conjugated molecular fragments can be useful in developing materials that exhibit singlet fission and thermally activated delayed fluorescence (TADF) for applications in highly efficient organic light-emitting diodes (OLEDs)^79,80^. Overall, this example illustrates the effectiveness of a dynamic-then-static ansatz^33^ coupled with NOSI for a balanced treatment of dynamic and static correlation involving CT states^26,27^. Furthermore, the M06–2X density functional is adequate to describe both local valence and inter-fragment CT states as well as their interactions.

### CT states of aryl-tetracyanoethene complexes

To further validate the performance of MAS-MSDFT@M06–2X, we examine the computed excitation energies of five bimolecular complexes that have a CT state as the lowest singlet excited state. These bimolecular complexes consist of one tetracyanoethene (TCNE) molecule and one aryl donor: benzene, toluene, *o*-xylene, naphthalene, and anthracene. This set of compounds has been used by Baer and coworkers to illustrate the optimization of system-specific range-separation parameters to improve the performance of TDDFT for CT states^17^, and by others to validate the performance of new density functionals^81^. In MSDFT, each bimolecular complex is partitioned into two monomer fragments, and the MAS includes a total of five local (monomer) valence excitations (four single excitations of the aromatic compounds involving HOMO, HOMO–1, LUMO, and LUMO + 1, plus one local $\pi \to \pi^{*}$ excitation of TCNE) and three CT states (HOMO and HOMO–1 of the aryl molecules to the LUMO of TCNE and one backward CT from TCNE to an aryl compound), along with the ground state. Although local valence excitations are included in the present calculation, there is little mixing with CT states, except in the narrow regions of (avoided) curve crossings along the direction of inter-fragment separation, if the inter-fragment distance is varied (not shown). Since we are not aware of prior computational results nor experimental data available for these valence excitations of the excimer complexes, both TDDFT and MSDFT results are given in Supplementary Table 1. Further, for these complexes, we have also used the hybrid PBE0 and B3LYP functionals with the cc-pVDZ and cc-pVTZ basis sets, and the results are given in Supplementary Table 3, which show similar performance as the M06–2X model. The molecular geometries used in the work of Stein et al. are adopted here^17^, and the BLE approach was used to optimize the fragmental block-localized Kohn–Sham orbitals of each configuration^30^.

The computed excitation energies are listed in Table 1, along with TDDFT results from the M06–2X and the CAM-QTP-02 functionals^81^, and the default and optimized range-separated BNL functional^17^ for comparison with experimental data^82–84^. Each of the aromatic compounds consists of two orbitals, HOMO and HOMO–1, close in energy (degenerate in benzene), resulting in two close-lying CT states from MSDFT and TDDFT calculations. In most complexes, only one broad peak was observed experimentally, but in some cases, two distinctively resolved peaks can be detected^82–84^. Thus, the average values of the two CT states are compared with experiments with a single resolvable peak, whereas two excited states in the naphthalene and anthracene complexes are used since they were resolved experimentally. Standard Kohn–Sham functionals severely underestimate the excitation energies of inter-fragment CT states, although the M06–2X functional exhibits smaller errors than B3LYP^17^. The default parameter for range-separation in the BNL functional overestimates the first CT excitation energies. However, after this parameter was specifically optimized for each complex to treat CT states, a good agreement with experiments (±0.2 eV) was obtained^17^. In the present study, by virtue of block-localization in MSDFT^30,32,33^, the limits of ionization potentials (IP) and electron affinities (EA) of each monomer at long inter-fragment separation are rigorously maintained—an important criterion for properly describing CT states. The performance of MSDFT on CT states for the molecular complexes is deemed sufficient because of a root-mean-square deviation (RMSD) of 0.12 eV and a mean signed error (MSE) of 0.08 eV between MSDFT@M06–2X/cc-pVDZ calculations and experiments for the seven resolvable CT states (Table 1). The use of a cc-pVTZ basis shows similar trends, but the energies are underestimated by about 0.1–0.2 eV (Supplementary Table 1). For comparison, TDDFT calculations using the latest version of ionization potential-fitted CAM-QTP-02 functional with the cc-pVTZ basis set gave MSE and RMSD errors of 0.21 and 0.34 eV for these compounds (without anthracene)^81^.

In the anthracene-TCNE complex, a second absorption peak was found in the UV range with transition energy of 2.79 eV in dichloromethane solution; however, its origin was uncertain and it was assumed to be due to CT from the HOMO–1 of anthracene to TCNE^84^. We found that the HOMO–1→TCNE CT transition occurs at a higher energy of 4.03 eV in the gas phase, which is separated from the first CT excitation by two local valence excitations (3.20 and 3.61 eV) of the anthracene molecule in the bimolecular complex. Even with the inclusion of solvent effects, estimated to be about 0.3 eV^17^, the reduction of the excitation energy is not sufficient to bring the second CT excitation down below 3 eV. Further, TDDFT using the M06–2X functional works very well for this complex, yielding CT energies of 1.89 and 3.55 eV, along with two valence excitations at 2.94 and 3.52 eV, consistent with MSDFT results (Supplementary Table 1). Therefore, the near UV band of the anthracene-TCNE complex observed experimentally^84^ can be assigned to a local valence excitation of the complex. Overall, the good agreement between MSDFT@M06–2X and experimental results suggests that the present MAS configurations are adequate for modeling CT states, which is further evaluated both on valence and CT states of the bimolecular complexes benchmark^2^.

### Local and charge-transfer states of bimolecular complexes

We next turn to a set of two-component molecular complexes that have been established using EOM-CCSDT or EOM-CCSDT-3 in ref. ^2^ (Table 2). In this dataset, each bimolecular complex is comprised of two different compounds from a list of nine molecules: F_2_, NH_3_, OF_2_, CH_2_ = CH_2_, CF_2_ = CH_2_, (CH_3_)_2_C = O, CH_3_NO_2_, pyrrole, and pyrazine. In MSDFT calculations, each bimolecular complex is divided into two monomer blocks as in the aryl-TCNE complexes. A MAS of locally excited and inter-fragment CT configurations is defined as follows: only single excitations involving key frontier orbitals (Supplementary Computational Procedure) of each monomer (1 → 1 and 2 → 2), called local excitations, are included along with one forward (1 → 2) and one backward (2 → 1) CT state. In particular, for NH_3_, CH_2_ = CH_2_, CF_2_ = CH_2_, and pyrrole, we include only HOMO → LUMO excitations, whereas both, $\text{n}\to \pi^{*}$ and $\pi \to \pi^{*}$ excitations are selected in acetone and nitromethane, noting that there are two $\text{n}\to \pi^{*}$ configurations in nitromethane from the in-phase and out-phase combinations of the oxygen lone pairs. For F_2_, all four $\pi \left(\pi_{\text{x}},\pi_{\text{y}},\pi_{\text{x}}^{\;*},\text{ and }\pi_{\text{y}}^{\;*}\right)\to \sigma^{*}$ excitations are used, and we adopted eight CSF for both pyrazine and OF_2_, due to excitations from two π and two lone pair orbitals, respectively, to two π* orbitals, and to two σ* orbitals. For the H_3_N…F_2_ complex, we also examined the effect of two double excitations of F_2_, but they only have a minimal effect (about 0.1 eV) on the computed excitation energies (Supplementary Table 2). These locally excited configurations are chosen to correspond to the valence states in the dataset of Kozma et al.^2,6,7^. Therefore, the largest number of excited configurations in the present MAS-MSDFT calculations is 14 for the pyrazine-F_2_ complex. All states in an MAS are spin adapted in this study. A full list of orbitals used to define these local single-excitations of all compounds are given in the Supplementary Computational Procedure.

Overall, for the fourteen CT states (Table 2) of the Kozma dataset, the mean error of MAS-MSDFT using the M06–2X functional is 0.06 eV and the RMSD is 0.19 eV relative to values determined by CCSDT(CCSDT-3). The excitation energies of CT states are slightly overestimated using MAS-MSDFT, but the overall agreement with results at the CCSDT level is good. Table 2 also includes 39 of the 41 local valence excited states in that dataset^2^, omitting two high-energy local excitations of TFE… ethene that are not represented in an MAS with just four con gurations in the present study. In comparison with EOM-CCSDT and EOM-CCSDT-3 results, the mean error for the local excitations of the bimolecular complexes is −0.12 eV and the RMSD is 0.48 eV. The somewhat large statistical error is clearly due to a systematic underestimate of the valence excitation energies of one compound, OF_2_ (Table 2)^2^. This deviation is due to the functional used which may be related to the particular optimization of the density functional. Without this complex, the mean and RMSD errors from the CCSDT (CCSDT-3) dataset are reduced to −0.04 and 0.29 eV, respectively. This agreement with EOM-CCSDT results is reasonable considering the simplicity of a small active space used in MSDFT calculations, and considering that this, in turn, enables a straightforward interpretation of adiabatic energies directly from its diabatic origin using Chirgwin–Coulson configuration weight.

### Significance of coupling between CT and valence excited states

A major difficulty in the study of CT states^2,6,7^ was its assignment from multi-configurational calculations. Often, visual inspection of natural orbitals of the difference density is made, but a set of numerical descriptors have recently been introduced on the basis of one-electron transition density^4,5^. In the latter analysis, the descriptor ω_CT_ specifies CT character in terms of the weight of contributions with charge separation in some predefined fragments. A ω_CT_ value of 0 corresponds to a local excitation, whereas a CT state is indicated by a value close to 1. ω_CT_ is very effective in cases where CT states can be clearly identified; however, about one third of the complexes in the benchmark set have strong mixing between a CT state and local excitations, making classification of CT state difficult^2,6^. Two bimolecular complexes, NH_3_…F_2_ and CF_2_ = CF_2_…CH_2_ = CH_2_, were especially highlighted in the work of Kozma et al.^2,6^ In the former complex, we included a second local excitation that was not listed in the original dataset (Table 1). In the latter complex, an inter-fragment separation of 5 Å (rather than its optimal geometry at 3.5 Å) was used to clearly separate ionic and covalent states. In these cases, the geometries at which avoided crossings occur and their energy gaps are sensitive to the level of theory used^6^.

**Fig. 2:**
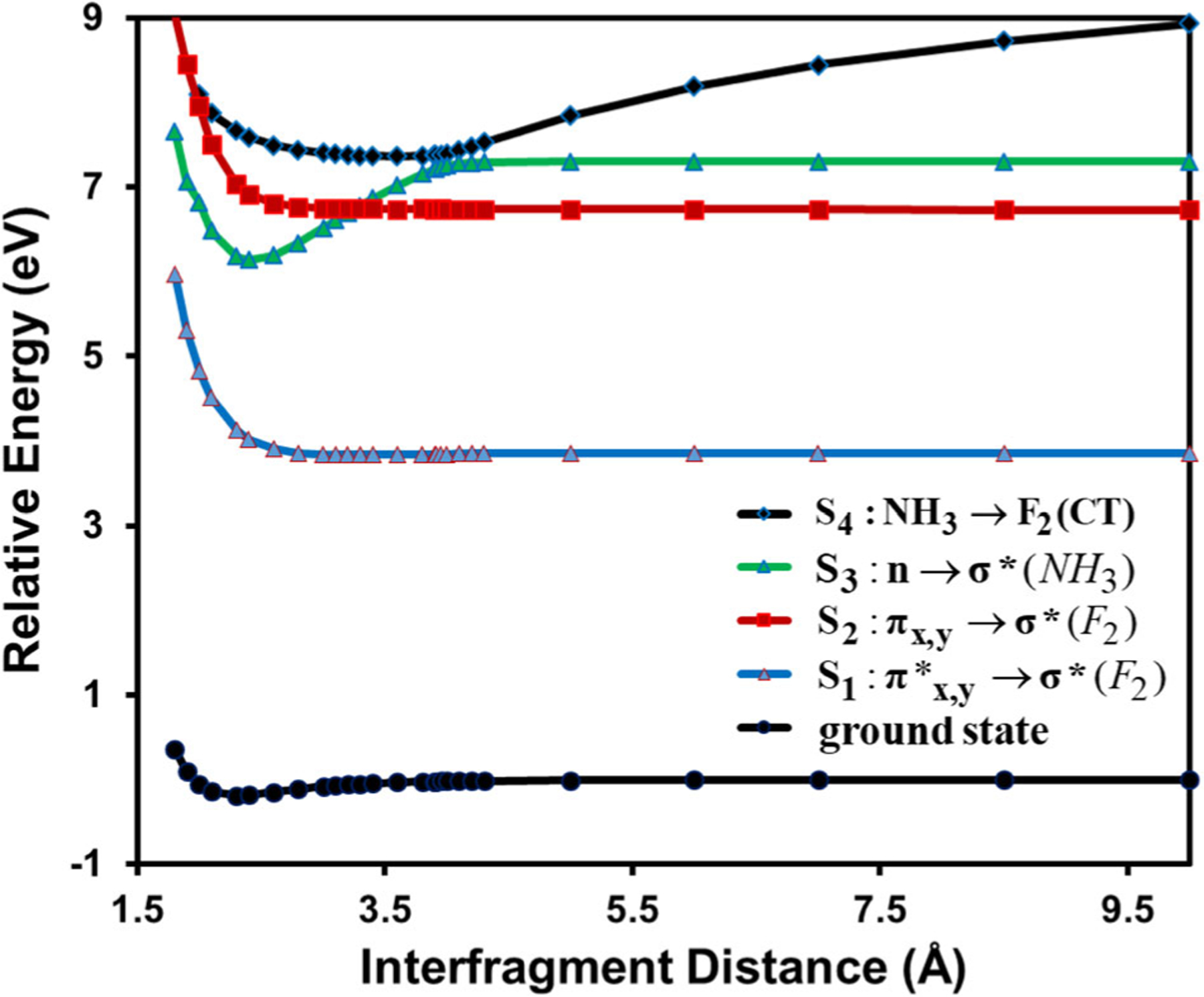
Potential energy curves of the adiabatic ground state, the first three singlet valence excited states, and the NH_3_ to F_2_ charge transfer state as a function of N-F distance (angstroms) of the NH_3_…F_2_ complex. All calculations are performed using MSDFT with the M06–2X functional and cc-pVDZ basis set and energies are given relative to the fully separated monomers. The first two excited states consist of two degenerate states and adiabatic states are labeled corresponding to those at long inter-fragment distances, which are different at short distances.

**Fig. 3:**
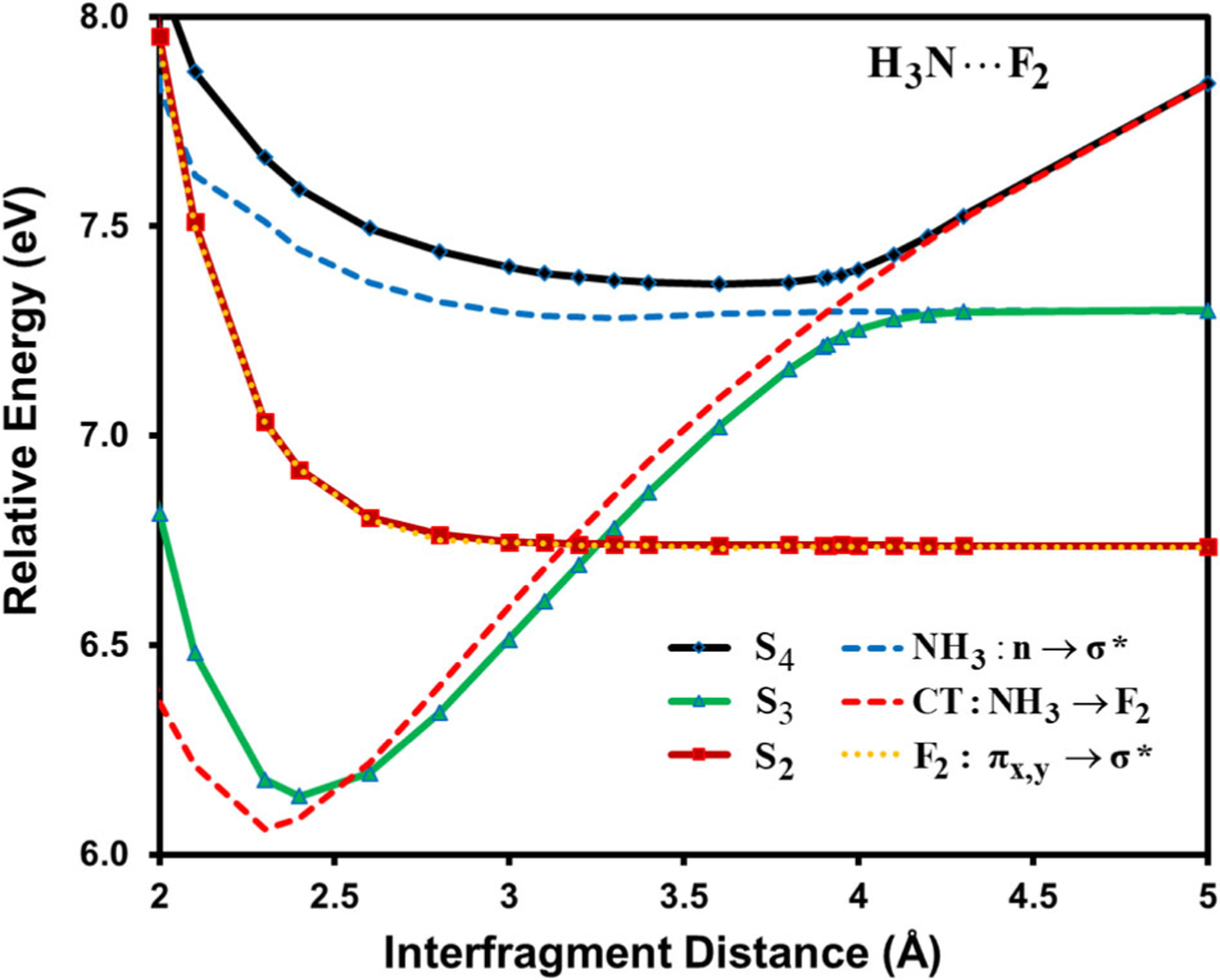
Potential energy curves of adiabatic (solid curves) and diabatic states (dotted curves) of NH_3_ → F_2_. The curve crossing region in Fig. 2 is displayed. Diabatic states are determined by using the generalized diabatic at construction (GDAC) method for the NH_3_ → F_2_ charge transfer and local transitions of n → σ* in NH_3_ and the ^1^Π_g_ state $\left(\pi_{\text{x},}\pi_{\text{y}}\to \sigma^{*}\right)$ in F_2_. Three adiabatic potential energy curves are shown as a function of N-F distance in the NH_3_…F_2_ complex.

In MSDFT, the basic configurations in the MAS are strictly localized diabatic and effective valence-bond (VB) states by construction^27,32,33^, whose contributions to an adiabatic state can be determined by Chirgwin–Coulson structural weights^41,42,85^, and the position of the diabatic state crossing point can be used to define the geometry of an avoided crossing between two interacting adiabatic states^24,86^. Fig. 2 shows the potential energy curves of the ground and the first four singlets excited states of NH_3_…F_2_ as a function of N…F distance. A clear feature of a CT state corresponding to NH_3_ to F_2_ transition can be observed by its strong dependence on inter-fragment separation, consistent with that reported previously^6^. In the region between 3 and 4 Å, the CT state crosses the 2^1^E state, a doubly degenerate local F_2_ excitation, and the 2^1^A_1_ state, a local excitation of ammonia, resulting in an allowed crossing at 3.3 Å and one avoided crossing of an energy gap of 0.14 eV. The corresponding orthogonalized diabatic states determined by the generalized diabatic at construction (GDAC) method^33^, along with the adiabatic curves, in the avoid crossing regions are depicted in Fig. 3, revealing the rapidly switching character of ionic and valence excited states. Interestingly, the N-F distance at the crossing point between the diabatic states corresponding to the CT state and the NH_3_ local excited state is located at 3.9 Å, slightly shorter than that (4.0 Å) of the smallest energy gap between the adiabatic curves. This may be compared with a value of about 3.75 Å determined using EOM-CCSDT^6^. Kozma et al. found that the second-order CC2 method yielded an unusually long crossing distance at about 5.3 Å with a tiny energy gap, while the non-iterative CCSD(T)(a)* method gives the same geometry as that of CCSDT, but the energy gap was underestimated for lack of a proper diagonalization^6^.

For the CT state of the NH_3_…F_2_ complex (CT1) at the optimal structure, the excitation energy of 6.64 eV (6.28 eV with cc-pVTZ basis) was assigned based on charge character analysis using CCSD results^2^. For comparison, the present MSDFT calculations yielded a value of 6.36 eV with a dominant CT structural weight of 0.96. The energy of the ^1^Π_g_ state of F_2_ (a π→σ* excitation) determined by using MSDFT@M06–2X is about 1 eV higher than that using CCSDT (ca. 5.7 eV), placing the CT state below that of the 2^1^E valence state. However, due to the mixing between CT and locally excited states in this case, the classification of a CT state is ambiguous, and the 2^1^E state was not listed in the dataset of Kozma et al.^2^. The lower energy value from CCSDT calculations was attributed to contributions from double excitation. However, the inclusion of HOMO to LUMO double excitations has little effects in the present MSDFT calculation (Supplementary Table 2). Nevertheless, a previous MRSDCI calculation predicted a value of 7.24 eV for this state, similar to the present calculation, and was assigned to a broadband of 6.5–7.5 eV from experiment^87^. This difference further highlights the sensitivity of state assignments; there is little uncertainty in assigning CT states in the present MSDFT approach since CT diabatic states are defined by construction in the MAS and they have well-defined configuration weights in each adiabatic state^85^.

**Fig. 4:**
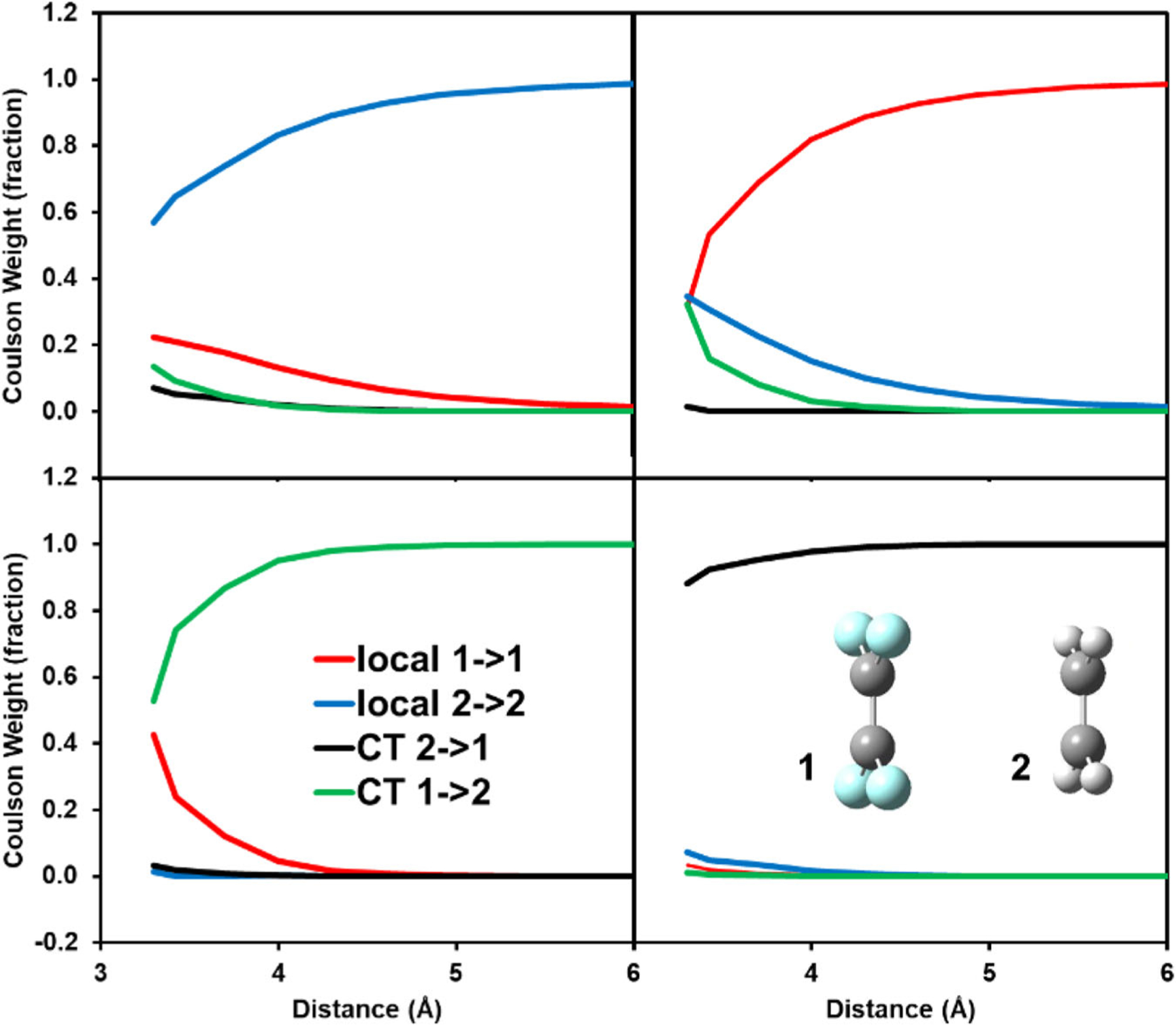
Chirgwin–Coulson structure weights of locally excited and charge transfer (CT) configurations. Shown in the top panel are the two lowest singlet valence excited states and the bottom figures exhibit two charge transfer states as a function of inter-fragment distance (Å) between tetrafluoroethene (**1**) and ethene (**2**). The M06–2X density functional and cc-pVTZ basis set are used in MSDFT calculations, including four configuration state functions.

For the tetrafluoroethene (TFE)…ethene complex, which was used as a test case for CT states in a number of previous studies^11,12,16,23,81^, It was not possible to clearly isolate the CT state in the optimal geometry since local excited states and CT states are strongly coupled^2^. Consequently, a structure at an interfragment separation of 5 Å was used, which shows a ω_CT_ value of 0.99. Figure 4 displays the Chirgwin–Coulson structure weights for the two lowest singlet states and the forward (ethene to TFE) and backward (TFE to ethene) CT states. The decrease of the dominant weight towards short distances in each of the four states shows a strong coupling between the locally excited π→π* resonance state and the ethene to TFE CT state at short distances. Strong mixing between local and CT states close to the optimal dimer geometry illustrates the difficulty to clearly assign a CT state using these structures^2^. Coupling interactions to CT states vary quickly with the change of inter-fragment distance; however, resonance delocalization interactions between the two π→π* states can persist at much longer separations (see the top row of graphs in Fig. 4). For the monomer species, we obtained vertical excitation energy of 8.2 and 7.5 eV for ethene and TFE (complex structure), which may be compared, respectively, with previous computational results of 8.4 eV from EOM-CCSDT-3^88^ and 7.0 eV from SAC-CI^89^, indicating that the present MAS is sufficient to model these low-lying states^19,30^.

It is of interest to comment on the three CT states of the pyrrole…pyrazine complexes. For the side-by-side hydrogen-bonded structure, the computed Chirgwin–Coulson structure weights provide a clear identification of the three CT states, CT8, CT9, and CT10 in Table 2, corresponding to nearly 100% contributions due to HOMO(pyrrole) → LUMO(Pyrazine), HOMO (pyrrole) → LUMO + 1(Pyrazine), and HOMO–1(pyrrole) → LUMO (Pyrazine) CT. Similarly, the ω_CT_ values were found in the range of 0.97 to 1.00 for these states^2,7^. However, in the π–π stacked configuration, strong resonance couplings among the CT states as well as covalent excited configurations are involved. The lowest ionic state CT11 (Table 2) is mixed with 24% of local n → π* valence excitations of pyrazine, and the CT component itself is a mixture between HOMO(pyrrole) → LUMO + 1(Pyrazine) and HOMO–1(pyrrole) → LUMO(Pyrazine) transitions. The next (local valence) excitation contains 23% ionic character. For comparison, the CT11 state has a ω_CT_ value of 0.84 from analysis of the EOM-CCSD results. The difference between the structure weight and ω_CT_ value could be due to a slight shift in geometry that has the largest coupling with CT state by different methods (MSDFT vs. CCSD) or could be the difference is the analysis method itself. MSDFT calculations show that CT12 and CT13 states are CT resonance states between HOMO(pyrrole) → LUMO + 1(Pyrazine) and HOMO–1(pyrrole) → LUMO(Pyrazine) configurations, plus a small amount of about 5% of local excitations. These two states are more than 95% CT character according to their Chirgwin–Coulson structure weights. However, transition density indices are ambiguous, showing just 61–66% of ionic character^2,7^.

In summary, the performance of MSDFT employing a MAS on local excited and inter-fragment CT states has been examined on a series of bimolecular complexes. MSDFT is a hybrid WFT and DFT, which follows a dynamic-then-static ansatz^36–38^. In MSDFT, dynamic correlation is first included via block-localized KS-DFT, and the orbitals are separately optimized for each determinant configuration. This is followed by a NOSI calculation to incorporate static correlation and to yield the adiabatic states^27^. Because of these two factors, it is expected that a small number of configurations would be sufficient to model low-lying local excited states and CT states^30,31^. In this work, we used a MAS consisting of singly excited configurations of key frontier orbitals plus forward and backward CT configurations. Using the Minnesota M06–2X density functional for each determinant configuration, we found that the present MAS-MSDFT method can provide a good description of CT states as well as the exciton resonance of local excited states. The computational cost of MSDFT is comparable to *M* separate KS-DFT optimizations (*M* is the number of determinants in an MAS), which can be performed independently in parallel, plus evaluation of the nonorthogonal off-diagonal matrix elements. Although the examples illustrated in this study featured separate monomers, a procedure similar to the projected hybrid orbital method for combined QM/MM calculations can be extended to treat fragments separated by covalent bonds. Due to the simplicity and interpretive power through energy decomposition analysis and determination of configuration weights^34^, the present method may be useful for direct dynamic simulations of nonadiabatic processes, and analyses of CT contributions to exciton coupling can be useful in the study of materials exhibiting TADF in future applications.

## METHODS

### Computational details

The anthracene monomer structure was optimized with the D_2h_ symmetry using Gaussian-16 at the B3LYP/aug-cc-pVTZ level of theory. Then, the monomer structure was kept fixed and used to construct face-to-face dimer configurations at a separation of 3.2 Å. Then, one monomer was slid along the long molecular axis up to 8 Å. Single-point energy calculations were performed using MSDFT, for which a total of 16 block-localized configurations (to form eight CSFs) plus the adiabatic ground state have been obtained using block-localized Kohn–Sham DFT. Geometries for the aryl-TCNE complexes were adopted from ref. ^17^, and the structures for the remaining bimolecular complexes are taken directly from the work of Kozma et al.^2^.

The computational procedure of MSDFT consists of two steps. First, a set of individually optimized determinant states are obtained to form a spin-adapted active space. Then, in the second step, NOSI is performed to yield the adiabatic ground and excited state energies. Here, we emphasize “state interaction” because on top of each determinant configuration, dynamic correlation is included via block-localized (constrained) KS-DFT. An analogy in WFT is a class of perturb-then-diagonalize methods. In particular, for local excitations of each monomer in a complex, the separately optimized open-shell singlet determinants with αβ and βα spin combinations were checked to converge to the same set of block-localized orbitals, and the orbitals were optimized using the method described in ref. ^30^. The spin-coupled singlet and triplet states (*M_s_* = 0) were obtained by switching the α and β electron spins of an open-shell configuration to yield a pair of spin-coupled determinants. The all-spin up triplet configuration (*M_s_* = 1) at each geometry was then obtained by a separate open-shell calculation. The energies for the *M_s_* = 1 and *M_s_* = 0 states were matched to yield the transition correlation energy contribution $E_{AB}^{\text{TDF}}$ (the TDF correlation) in spin-pair coupling interactions according to Eq 3 ^28,65^.

The energies for the diabatic states were determined using the GDAC method^33^. The Minnesota M06–2X density functional along with the cc-pVDZ basis functions are used in all calculations except that for the aryl-TCNE complexes where the PBE0 and B3LYP functionals and the cc-pVTZ basis set were also used. Additional details are summarized in the Supplementary Information.

The MSDFT method described in this paper has been implemented in a locally modified version of the GAMESS-US program^90^, with which all calculations have been performed.

## Supplementary Material

Supporting Info

## Figures and Tables

**Table 1.** Computed first and second excitation energies (eV) of aryl-tetracyanoethene complexes in the gas phase along with experimental data.

Aryl-TCNE complex	M06–2X	BNL^a^	BNL(γopt)^a^	CAM-QTP-02^b^	MSDFT @M06–2X	Expt.^c^
Benzene	2.99, 3.04	4.4	3.8	3.85	3.66, 3.74	3.59 (3.67)
Toluene	2.74, 2.94	4.0	3.4	3.50	3.28, 3.63	3.36 (3.35)
*o*-Xylene	2.48, 2.78	3.7	3.0	3.25	3.07, 3.32	3.15 (3.15)
Naphthalene	1.94, 2.73	3.3	2.7	2.80, 3.57	2.69, 3.43	2.60, 3.23
Anthracene	1.89, 2.94	2.6	2.1		1.62, 2.90 3.73^d^	1.73, 2.79^e^

a ref. ^17^.

b ref. ^81^.

c ref. ^82^ and data in parentheses from ref. ^83^.

d Values reduced by 0.3 eV for solvent effects estimated in ref. ^17^.

e CH2Cl2 solution, ref. ^84^.

**Table 2.** Excitation energies (eV) of local (1 → 1 or 2 → 2) and charge transfer (1 → 2 or 2 → 1) states computed using EOM-CCSDT (EOM-CCSDT-3 for acetone-nitromethane and pyrrole-pyrazine complexes) and MAS-MSDFT using the M06–2X functional, along with charge character (ω_CT_) determined at the EOM-CCSD level and Chirgwin–Coulson structure weight (W_CT_) of charge transfer configuration from MSDFT.

Complex (1…2) and CT state	excitation	State^a^	EOM-CCSDT^b^	MSDFT	ω_CT_^c^	W_CT_
H_3_N…F_2_	2 → 2	1^1^E	3.97	4.31		
CT1	1 → 2(1)	2^1^A_1_	6.64	6.37	0.76	0.96
	2 → 2	2^1^E	NA	7.22		
	1 → 1	3^1^A_1_	7.98	7.82		
	2 → 1	BCT		15.7		1.00
Me_2_CO…F_2_	1 → 1	2 A″	4.39	4.30		
	2 → 2	2 A′	4.18	4.42		
	2 → 2	1 A″	4.19	4.43		
CT2	1 → 2	3 A″	5.85	5.83	0.96	1.00
	2 → 1	BCT		14.1		1.00
Pyrazine…F_2_	2 → 2	2 A_1_	4.16	4.41		
	2 → 2	2B_1_	4.19	4.42		
	1 → 1	3A_1_	4.28	4.28		
	1 → 1	2B_1_	4.99	4.48		
	1 → 1	1 A_2_	5.04	5.26		
	1 → 1	1B_2_	5.81	5.69		
CT3	1 → 2	2B_2_	6.28	6.27	0.98	1.00
CT4	2 → 1(2)	2 A_2_	6.45	6.32	0.64	0.97
H_3_N…OF_2_	2 → 2	2 A′	4.20	3.42		
	2 → 2	1 A″	4.93	4.85		
	2 → 2	2 A″	6.64	5.01		
	2 → 2	3 A′	6.83	5.36		
CT5	1 → 2(1)	4 A′	7.04	7.07	0.86	0.92
	2 → 2	3 A″	7.11	7.97		
	1 → 1(2)	5 A′	7.96	7.62	0.04	0.08
	2 → 1	BCT		13.6		1.00
Me_2_CO… MeNO_2_	2 → 2	2 A	4.04	4.18		
	1 → 1	3 A	4.42	4.48		
	2 → 2	4 A	4.43	5.05		
CT6	1(2) → 2	5 A	6.42	6.51	0.80	0.94
	2(1) → 2	6 A	6.58	6.33	0.20	0.06
	2 → 1	BCT		9.48		
Pyrazine… NH_3_	1 → 1	2 A′	4.35	4.26		
	1 → 1	1 A″	4.99	4.56		
	1 → 1	2 A″	5.08	5.30		
	1 → 1	3 A′	5.88	5.68		
	1 → 1	3 A″	6.83	6.49		
	1 → 1	4 A′	7.04	7.03		
CT7	2 → 1(2)	5 A′	7.49	7.72	0.63	0.71
	2 → 2(1)	6 A′	7.80	7.32	0.27	0.29
	2 → 1(L + 1)	CT		8.37		1.00
	1 → 2	BCT		9.89		1.00
H-bonded						
Pyrrole-pyrazine	2 → 2	1B_2_	4.38	4.38		
	2 → 2	1B_1_	5.01	4.59		
	2 → 2	1A_2_	5.22	5.42		
CT8	1 → 2	2B_1_	5.27	5.53	1.00	1.00
CT9	1 → 2	2 A_1_	6.00	6.39	0.97	1.00
	2 → 2	2B_2_	6.01	5.74		
CT10	1 → 2	3 A_1_	6.17	6.42	0.99	1.00
	1 → 1	4A_1_	6.50	6.70		
	2 → 1	BCT		11.3		1.00
Stacked						
Pyrrole-pyrazine	2 → 2	2 A′	4.33	4.25		
	2 → 2	3 A′	4.98	4.49		
	2 → 2	1 A″	5.09	5.22		
CT11	1(2) → 2	2 A″	5.48	5.33	0.84	0.76
	2(1) → 2	3 A″	5.90	5.83		0.23
CT12	1(2) → 2(1)	4 A′	6.07	6.19	0.66	0.86, 0.09
CT13	2(1) → 1(2)	5 A′	6.34	6.55	0.61	0.87, 0.09
TFE…ethene (5 Å)	1(2) → 1(2)	1B_2_	7.43	7.60		
	2(1) → 2(1)	1B_1_	8.61	8.14		
CT14	1 → 2	5B_1_	10.57	10.40	0.99	1.00
	2 → 1	BCT		10.68		1.00

a *BCT* backward charge transfer relative to the direction of CT states in the first column.

b Excitation energies were determined for all complexes using EOM-CCSDT, except acetone-nitromethane and pyrrole-pyrazine complexes for which EOM-CCSDT-3 was used^2^.

c Determined at the EOM-CCSD level of theory.

The parentheses in the “excitation” column denote minor contributions from the other monomer to the initial and/or final orbitals for states of mixed local and CT character. The set of 14 CT states in reference 2 are specifically indicated in the first column.

## Data Availability

The data that support the plots within this paper are available from the corresponding authors upon request.
