# Supplementary material for: Minimal-active-space multistate density functional theory for excitation energy involving local and charge transfer states
Source: NPJ Comput Mater. Author manuscript; Available in PMC 2023 Jan 27. (PMC9881008; doi:10.1038/s41524-021-00624-3)
Supplement: Supporting Info [file NIHMS1815446-supplement-Supporting_Info.pdf]

Supplementary Information for

Minimal-Active-Space Multistate Density Functional Theory for Excitation Energy Involving Local and Charge Transfer States

Ruoqi Zhao,[1,2] Christian P. Hettich,[3] Xin Chen,[1,4]* and Jiali Gao[1,3,4]*

1. Institute of Systems and Physical Biology, Shenzhen Bay Laboratory, Shenzhen 518055, China

2. Institute of Theoretical Chemistry, Jilin University, Changchun, Jilin Province 130023, China

3. Department of Chemistry and Supercomputing Institute, University of Minnesota, Minneapolis, Minnesota 55455, United States

4. Beijing University Shenzhen Graduate School, Shenzhen 518055, China

This document contains a summary of the computational details of minimal-active-space multistate density functional theory (MS-MSDFT) calculations for charge transfer states and local valence excitations of bimolecular complexes, plus a list of active orbitals for each monomer structure (16 pages).

Computational procedure of the MAS-MSDFT method.

1. *Ground state optimization*. First, the molecular orbitals of the A…B complex is partitioned into two fragment-block localized orbitals (BLO), which are formed using only the basis orbitals located on atoms of each monomer.[1] The ground state is optimized using block-localized Kohn-Sham density functional theory (KS-DFT),[2] resulting in the KS-determinant $\Psi_{AB} = |AB> = \hat{A}\{(\Omega_A)(\Theta_B)\}$ where (Ω_A) and (Θ_B) are products of occupied block-localized Kohn-Sham orbitals of monomer A and B, respectively. The coefficient matrix of Ψ_{AB} is A-B block diagonal. Throughout this study, the Minnesota M06-2X functional and cc-pVDZ basis set (except a few cases done with cc-pVTZ) are used.
2. *Selection of active orbitals*. In the present study involving low-lying local excitations and cross fragment charge transfer (CT) states, the highest occupied molecular orbitals (HOMO) and the lowest unoccupied molecular orbitals (LUMO) of fragment A and B, and one or two additional high- π or n (lone pair) occupied orbitals of π or n (lone pair) types are included to match the states in the Kozma dataset.[3,4] In the case of pyrazine, LUMO+1, a π* orbitals, is also included.
3. *Local valence excited states*. Excited configurations in the active space for each bimolecular complex A…B include only singly excitations, local within the monomer space. Therefore, these singly excitations can be collectively expressed by the $|A^*B>$ type and the $|AB^*>$ type, corresponding to singly excited determinants $\Psi(A_i^a B) = \hat{A}\{(\Omega_A)_i^a(\Theta_B)\}$ and $\Psi(AB_j^b) = \hat{A}\{(\Omega_A)(\Theta_B)_j^b\}$.[5] Here, $(\Omega_A)_i^a$ indicates that the occupied orbital product is switching occupied orbital *i* with virtual orbital *a* of monomer A, both of which in the selected orbital space in step (2). Similarly, $(\Theta_B)_j^b$ denotes a product of occupied orbitals in which the occupied orbital *j* is swapped with virtual orbital *b* of monomer B.
4. *Charge transfer states*. Charge transfer (CT) states are included, typically corresponding to excitation from the HOMO (highest occupied molecular orbital) of one monomer to the LUMO (lowest unoccupied molecular orbital) of the other monomer. The CT from the monomer with a lower ionization potential is called forward charge transfer, e.g., $\Psi_{FCT}(A^+B^-) = \hat{A}\{(\Omega_A)_i(\Theta_B)^b\}$, and the reverse direction of CT is called backward charge transfer $\Psi_{BCT}(A^-B^+) = \hat{A}\{(\Omega_A)^a(\Theta_B)_j\}$.[5] In the case of pyrrole…pyrazine complex, the HOMO-1 of pyrrole to the LUMO of pyrazine, and HOMO of pyrrole to the LUMO+1 of pyrazine CT configurations are also included, because these CT state are close in energy.
5. *Optimization of excited configurations and NOCI.* Each local singly excited determinant and charge transfer state is individually optimized using the block-localized excitation (BLE) method.[5] All calculations were performed using unrestricted block-localized KS-DFT, and the sets of optimized α and β spin orbitals are switched and paired to produce a singlet configuration state function (CSF) and also the corresponding $M_s = 0$ component of the triplet state.[6,7] These CSFs forms the minimal active space in MSDFT

nonorthogoanl configuration interaction (NOCI) calculations to yield the adiabatic states discussed in the text.[5,8]

Transition density functional for the off-diagonal, diabatic coupling elements.

The transition density functional, a novel class of correlation functional that does not exist in Kohn-Sham DFT, needed to define the electronic coupling to yield the singlet configuration state functions is based on a procedure proposed by Ziegler et al. in a different context.[7] Given that the high spin (triplet in the present application) configuration of an open-shell system can be adequately treated by a single determinant in KS-DFT, we enforce the energy degeneracy with the lower spin component represented by the CSF of the high-spin state, resulting from exactly the same spin-coupling interaction that produces the low-spin CSF (singlet in this study).[6]

For all other diabatic coupling interactions, we used the overlap-scaled correlation energy of the two interacting Kohn-Sham determinants to approximate the value of the transition density functional.[2,6,8]

Active orbitals.

The "active orbitals" for each molecular fragment is listed below. In the dimer calculation, the molecular blocks are kept, but the ground state BL-KS-DFT optimization includes mutual polarization effects, but interfragment charge transfer is prevented due to block-localization constraint.[9] CT effects are specifically defined as described above. All configurations in the active space used in MSDFT calculations are diabatic states that have strict asymptotic limits that can be interpreted.[10,11] Thus, the procedure yields diabatic states by construction, which can be used to evaluate Chirgwin-Coulson structural weights[12] to assess the nature of an adiabatic state in terms of its CT character. The following lists the orbitals and qualitative orbital levels and their occupations of each monomer for block-localized excitations in bimolecular complexes.

F_2

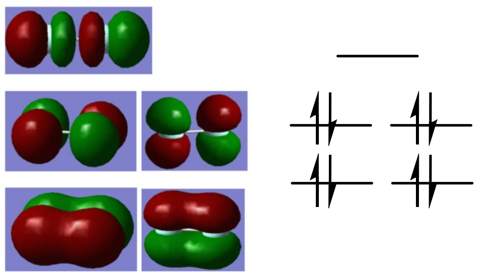

NH_3

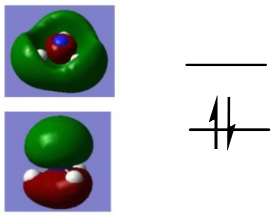

$CH_2{=}CH_2$

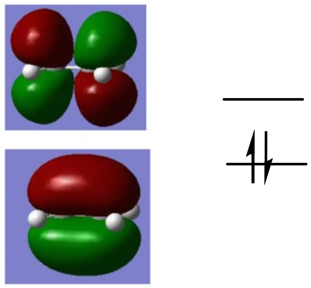

$CF_2{=}CF_2$

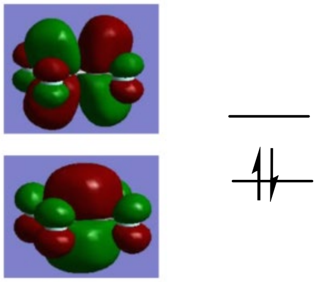

$CH_2{=}O$

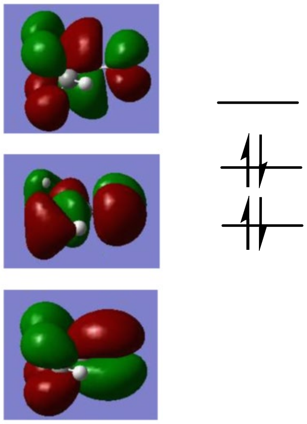

CH_3NO_2

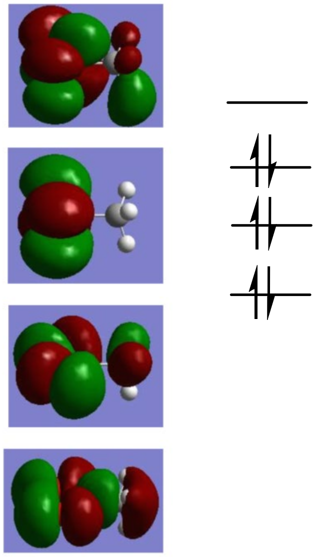

Pyrazine

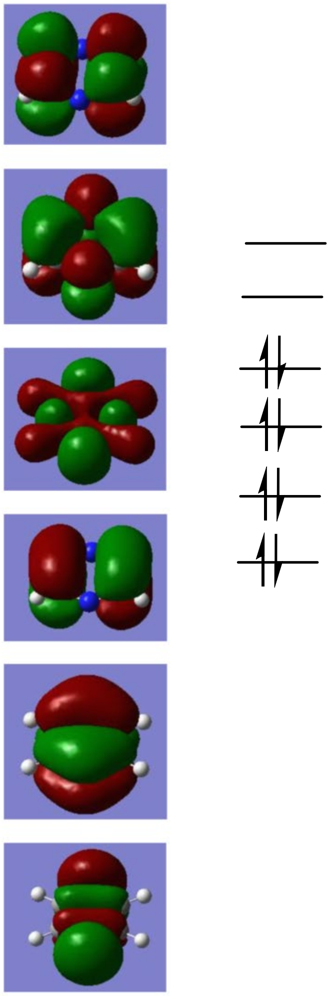

Pyrrole

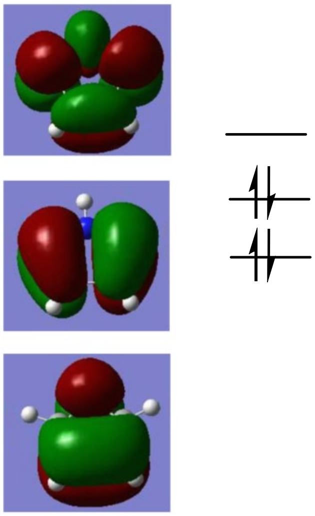

Table S1. Computed excitation energies (eV) for the first 5 singlet states using TDDFT and MSDFT along with M06-2X/cc-pVDZ.

	TDDFT@M06-2X/cc-pVDZ					MSDFT@M06-2X/cc-pVDZ				
aryl-TCNE	1	2	3	4	5	1	2	3	4	5
benzene	2.99*	3.04*	4.63	4.87	5.25	3.66*	3.74*	4.41	4.60	5.52
toluene	2.74*	2.94*	4.63	4.88	5.25	3.28*	3.63*	4.61	4.70	5.00
o-xylene	2.48*	2.78*	4.61	4.89	5.24	3.07*	3.32*	4.48	4.65	5.52
naphthelene	1.94*	2.73*	4.06	4.59	4.67	2.69*	3.43*	3.87	4.19	4.62
anthracene	1.89*	2.94	3.52	3.55*	4.11	1.92*	3.20	3.61	4.03*	4.56

* Charge transfer state.

Table S2

Complex (1…2)	excitation	State[a]	EOM-CCSDT	MSDFT(SD)	MSDFT(S)	W_{CT}
$H_3N…F_2$	2→2	1^1E	3.97	4.42	4.41	
CT1	1→2(1)	2^1A_1	6.64	6.45	6.46	0.96
	2→2	2^1E	n/a	7.33	7.32	
	1→1	3^1A_1	7.98	7.96	7.95	
	2→1	BCT		15.7	15.8	1.00

Table S3. Computed First and Second Excitation Energies (eV) of Several Gas Phase Ar-TCNE Systems using MSDFT with the hybrid PBE0 and B3LYP functionals and cc-pVDZ and cc-pVTZ basis sets. The corresponding experimental values are also listed with values in parentheses from different experiments (see main text).

Ar	b3lyp cc-pVTZ	b3lyp cc-pVDZ	PBE0 cc-pVTZ	PBE0 cc-pVDZ	Exp.
benzene	3.33,3.37	3.55,3.58	3.28,3.32	3.48,3.5	3.59 (3.67)
toluene	2.97,3.21	3.17,3.42	2.92,3.16	3.11,3.23	3.36 (3.35)
xylene	2.66,2.96	2.87,3.17	2.61,2.91	2.81,2.97	3.15 (3.15)
naphthalene	2.15,2.9	2.38,3.02	2.15,2.9	2.35,3.11	2.60, 3.23
anthracene	1.75,2.8	1.89,2.8	1.74,2.82	1.88,2.72	1.73, 2.79

References

1 Mo, Y., Gao, J. & Peyerimhoff, S. D. Energy decomposition analysis of intermolecular interactions using a block-localized wave function approach. *J. Chem. Phys.* **112**, 5530-5538 (2000).

2 Cembran, A., Song, L., Mo, Y. & Gao, J. Block-localized density functional theory (BLDFT), diabatic coupling, and its use in valence bond theory for representing reactive potential energy surfaces. *J. Chem. Theory Comput.* **5**, 2702-2716.. (2009).

3 Kozma, B. *et al.* A New Benchmark Set for Excitation Energy of Charge Transfer States: Systematic Investigation of Coupled Cluster Type Methods. *J. Chem. Theory Comput.* **16**, 4213-4225, doi:10.1021/acs.jctc.0c00154 (2020).

4 Tajti, A., Kozma, B. & Szalay, P. G. Improved Description of Charge-Transfer Potential Energy Surfaces via Spin-Component-Scaled CC2 and ADC(2) Methods. *J. Chem. Theory Comput.* **17**, 439-449, doi:10.1021/acs.jctc.0c01146 (2021).

5 Bao, P., Hettich, C. P., Shi, Q. & Gao, J. Block-Localized Excitation for Excimer Complex and Diabatic Coupling. *J. Chem. Theory Comput.* **17**, 240-254, doi:10.1021/acs.jctc.0c01015 (2021).

6 Grofe, A., Chen, X., Liu, W. J. & Gao, J. L. Spin-Multiplet Components and Energy Splittings by Multistate Density Functional Theory. *J. Phys. Chem. Lett.* **8**, 4838-4845, doi:10.1021/acs.jpclett.7b02202 (2017).

7 Ziegler, T., Rauk, A. & Baerends, E. J. Calculation of Multiplet Energies by Hartree-Fock-Slater Method. *Theor. Chim. Acta* **43**, 261-271, doi:Doi 10.1007/Bf00551551 (1977).

8 Gao, J., Grofe, A., Ren, H. & Bao, P. Beyond Kohn–Sham Approximation: Hybrid Multistate Wave Function and Density Functional Theory. *J. Phys. Chem. Lett.* **7**, 5143-5149 (2016).

9 Mo, Y. R., Bao, P. & Gao, J. L. Energy decomposition analysis based on a block-localized wavefunction and multistate density functional theory. *Phys Chem Chem Phys* **13**, 6760-6775, doi:Doi 10.1039/C0cp02206c (2011).

10 Grofe, A., Qu, Z. X., Truhlar, D. G., Li, H. & Gao, J. L. Diabatic-At-Construction Method for Diabatic and Adiabatic Ground and Excited States Based on Multistate Density Functional Theory. *J. Chem. Theory Comput.* **13**, 1176-1187, doi:10.1021/acs.jctc.6b01176 (2017).

11 Liu, M., Chen, X., Grofe, A. & Gao, J. Diabatic States at Construction (DAC) through Generalized Singular Value Decomposition. *J Phys Chem Lett* **9**, 6038-6046, doi:10.1021/acs.jpclett.8b02472 (2018).

12 Chirgwin, H. B. & Coulson, C. A. *Proc. R. Soc. London Ser. A* **2**, 196. (1950).

The H3N…F2 bimolecular complex at an N-F distance of about 3.6 angstroms is used to further illustrate the computational details. All computations were performed using M06-2X functional or Kohn-Sham determinants and the cc-pVDZ basis set.

Coordinates for the complex.

1	9	0.007371	-0.078831	-0.850309
2	9	0.020214	-0.216134	-2.331342
3	7	-0.023784	0.254241	2.742390
4	1	-0.114228	1.223066	3.058937
5	1	-0.793879	-0.249671	3.189641
6	1	0.826328	-0.098394	3.189554

MAS determinant basis configurations: 4 SA single excitations of F_2 and 1 SA single excitation of NH_3, 1 forward and 1 backward (two possible, but they are high in energy. so only one included), plus one block-localized ground state configuration.

1. $\Psi_1 = \hat{A}\{[\Omega_{F_2}^{core}\pi_x^2\pi_y^2(\pi_x^*)^2(\pi_y^*)_\alpha^1(\sigma^*)_\beta^{\bar{1}}][\Theta_{NH_3}]\}$
2. $\Psi_2 = \hat{A}\{[\Omega_{F_2}^{core}\pi_x^2\pi_y^2(\pi_x^*)^2(\pi_y^*)_\beta^{\bar{1}}(\sigma^*)_\alpha^1][\Theta_{NH_3}]\}$
3. $\Psi_3 = \hat{A}\{[\Omega_{F_2}^{core}\pi_x^2\pi_y^2(\pi_y^*)^2(\pi_x^*)_\alpha^1(\sigma^*)_\beta^{\bar{1}}][\Theta_{NH_3}]\}$
4. $\Psi_4 = \hat{A}\{[\Omega_{F_2}^{core}\pi_x^2\pi_y^2(\pi_y^*)^2(\pi_x^*)_\beta^{\bar{1}}(\sigma^*)_\alpha^1][\Theta_{NH_3}]\}$
5. $\Psi_5 = \hat{A}\{[\Omega_{F_2}^{core}\pi_x^2(\pi_x^*)^2(\pi_y^*)^2(\pi_y)_\alpha^1(\sigma^*)_\beta^{\bar{1}}][\Theta_{NH_3}]\}$
6. $\Psi_6 = \hat{A}\{[\Omega_{F_2}^{core}\pi_x^2(\pi_x^*)^2(\pi_y^*)^2(\pi_y)_\beta^{\bar{1}}(\sigma^*)_\alpha^1][\Theta_{NH_3}]\}$
7. $\Psi_7 = \hat{A}\{[\Omega_{F_2}^{core}\pi_y^2(\pi_x^*)^2(\pi_y^*)^2(\pi_x)_\alpha^1(\sigma^*)_\beta^{\bar{1}}][\Theta_{NH_3}]\}$
8. $\Psi_8 = \hat{A}\{[\Omega_{F_2}^{core}\pi_y^2(\pi_x^*)^2(\pi_y^*)^2(\pi_x)_\beta^{\bar{1}}(\sigma^*)_\alpha^1][\Theta_{NH_3}]\}$
9. $\Psi_9 = \hat{A}\{[\Omega_{F_2}][\Theta_{NH_3}^{core}n_\alpha^1(\sigma^*)_\beta^{\bar{1}}]\}$
10. $\Psi_{10} = \hat{A}\{[\Omega_{F_2}][\Theta_{NH_3}^{core}n_\beta^{\bar{1}}(\sigma^*)_\alpha^1]\}$
11. $\Psi_{11} = \hat{A}\{[\Omega_{F_2}(\sigma^*)_\alpha^1]^-[\Theta_{NH_3}^{core}n_\beta^{\bar{1}}]^+\}$
12. $\Psi_{12} = \hat{A}\{[\Omega_{F_2}(\sigma^*)_\beta^{\bar{1}}]^-[\Theta_{NH_3}^{core}n_\alpha^1]^+\}$
13. $\Psi_{13} = \hat{A}\{[(\Omega_{F_2}^{core}\pi_x^2\pi_y^2(\pi_x^*)^2(\pi_y^*)_\alpha^1]^+[\Theta_{NH_3}(\sigma^*)_\beta^{\bar{1}}]^-\}$
14. $\Psi_{14} = \hat{A}\{[(\Omega_{F_2}^{core}\pi_x^2\pi_y^2(\pi_x^*)^2(\pi_y^*)_\beta^{\bar{1}}]^+[\Theta_{NH_3}(\sigma^*)_\alpha^1]^-\}$
15. $\Psi_{15} = \hat{A}\{[\Omega_{F_2}][\Theta_{NH_3}]\}$

H and S matrices using optimized BL-Kohn-Sham determinants

15	1		
1	1	1.000000000000	-254.756941054607
1	2	0.000000318758	0.029019081109
1	3	0.000093648542	-0.023857892565
1	4	0.000000008993	0.000000431012
1	5	-0.027859628655	7.099134846228
1	6	0.000000000510	-0.000740223753
1	7	-0.000100156772	0.025516487830
1	8	-0.000000002313	-0.000002278442

1	9	-0.000000986245	0.000253632790
1	10	-0.000000654705	0.000169212685
1	11	0.000002901012	-0.000741756108
1	12	0.000002753624	-0.000704069610
1	13	0.004912623856	-1.253724342774
1	14	0.000000000121	0.000046249978
1	15	-0.000620546139	0.158157988733
2	1	0.000000318758	0.029019081109
2	2	1.000000000000	-254.756941054607
2	3	0.000000008993	0.000000431012
2	4	0.000093648542	-0.023857892565
2	5	0.000000000510	-0.000740223753
2	6	-0.027859628655	7.099134846228
2	7	-0.000000002313	-0.000002278442
2	8	-0.000100156772	0.025516487830
2	9	-0.000000654705	0.000169212685
2	10	-0.000000986245	0.000253632790
2	11	0.000002753624	-0.000704069610
2	12	0.000002901012	-0.000741756108
2	13	0.000000000121	0.000046249978
2	14	0.004912623856	-1.253724342774
2	15	-0.000620546139	0.158157988733
3	1	0.000093648542	-0.023857892565
3	2	0.000000008993	0.000000431012
3	3	1.000000000000	-254.757061157585
3	4	0.000000000014	0.029081129828
3	5	-0.000113731264	0.028972994234
3	6	-0.000000000064	-0.000003345478
3	7	-0.033319516324	8.490413674192
3	8	-0.000000000024	-0.000796445116
3	9	-0.000000037819	0.000009649281
3	10	-0.000000037179	0.000009534862
3	11	-0.000000082891	0.000021178112
3	12	0.000000026510	-0.000006812176
3	13	-0.003927085588	1.002213251301
3	14	0.000000000009	-0.000036724649
3	15	-0.000056276500	0.014337024224
4	1	0.000000008993	0.000000431012
4	2	0.000093648542	-0.023857892565
4	3	0.000000000014	0.029081129828
4	4	1.000000000000	-254.757061157585
4	5	-0.000000000064	-0.000003345478
4	6	-0.000113731264	0.028972994234
4	7	-0.000000000024	-0.000796445116
4	8	-0.033319516324	8.490413674192
4	9	-0.000000037179	0.000009534862
4	10	-0.000000037819	0.000009649281
4	11	0.000000026510	-0.000006812176
4	12	-0.000000082891	0.000021178112
4	13	0.000000000009	-0.000036724649
4	14	-0.003927085588	1.002213251301
4	15	-0.000056276500	0.014337024224
5	1	-0.027859628655	7.099134846228
5	2	0.000000000510	-0.000740223753
5	3	-0.000113731264	0.028972994234
5	4	-0.000000000064	-0.000003345478

5	5	1.000000000000	-254.626835843113
5	6	0.000000000014	0.026032249477
5	7	0.000087610447	-0.022307683768
5	8	0.000000000058	0.000002384373
5	9	-0.000000007091	0.000001828632
5	10	-0.000000004741	0.000001225923
5	11	0.000000040978	-0.000010478230
5	12	-0.000000060746	0.000015495261
5	13	-0.001524981480	0.388832524387
5	14	0.000000000004	0.000029129707
5	15	-0.000002230361	0.000572269127
6	1	0.000000000510	-0.000740223753
6	2	-0.027859628655	7.099134846228
6	3	-0.000000000064	-0.000003345478
6	4	-0.000113731264	0.028972994234
6	5	0.000000000014	0.026032249477
6	6	1.000000000000	-254.626835843113
6	7	0.000000000058	0.000002384373
6	8	0.000087610447	-0.022307683768
6	9	-0.000000004741	0.000001225923
6	10	-0.000000007091	0.000001828632
6	11	-0.000000060746	0.000015495261
6	12	0.000000040978	-0.000010478230
6	13	0.000000000004	0.000029129707
6	14	-0.001524981480	0.388832524387
6	15	-0.000002230361	0.000572269127
7	1	-0.000100156772	0.025516487830
7	2	-0.000000002313	-0.000002278442
7	3	-0.033319516324	8.490413674192
7	4	-0.000000000024	-0.000796445116
7	5	0.000087610447	-0.022307683768
7	6	0.000000000058	0.000002384373
7	7	1.000000000000	-254.626830676823
7	8	0.000000000005	0.026034195781
7	9	0.000000198986	-0.000051030108
7	10	0.000000145414	-0.000037389345
7	11	-0.000000102692	0.000026257292
7	12	0.000000025182	-0.000006405952
7	13	0.001215555874	-0.309936829626
7	14	-0.000000000015	-0.000023220796
7	15	0.000109442366	-0.027869780779
8	1	-0.000000002313	-0.000002278442
8	2	-0.000100156772	0.025516487830
8	3	-0.000000000024	-0.000796445116
8	4	-0.033319516324	8.490413674192
8	5	0.000000000058	0.000002384373
8	6	0.000087610447	-0.022307683768
8	7	0.000000000005	0.026034195781
8	8	1.000000000000	-254.626830676823
8	9	0.000000145414	-0.000037389345
8	10	0.000000198986	-0.000051030108
8	11	0.000000025182	-0.000006405952
8	12	-0.000000102692	0.000026257292
8	13	-0.000000000015	-0.000023220796
8	14	0.001215555874	-0.309936829626
8	15	0.000109442366	-0.027869780779

9	1	-0.000000986245	0.000253632790
9	2	-0.000000654705	0.000169212685
9	3	-0.000000037819	0.000009649281
9	4	-0.000000037179	0.000009534862
9	5	-0.000000007091	0.000001828632
9	6	-0.000000004741	0.000001225923
9	7	0.000000198986	-0.000051030108
9	8	0.000000145414	-0.000037389345
9	9	1.000000000000	-254.613821398558
9	10	0.000530318807	-0.121968417873
9	11	0.006862612483	-1.752327181270
9	12	0.000100745924	-0.025729114769
9	13	0.000000004209	-0.000001079883
9	14	0.000000001711	-0.000000433362
9	15	0.000673909075	-0.175740613655
10	1	-0.000000654705	0.000169212685
10	2	-0.000000986245	0.000253632790
10	3	-0.000000037179	0.000009534862
10	4	-0.000000037819	0.000009649281
10	5	-0.000000004741	0.000001225923
10	6	-0.000000007091	0.000001828632
10	7	0.000000145414	-0.000037389345
10	8	0.000000198986	-0.000051030108
10	9	0.000530318807	-0.121968417873
10	10	1.000000000000	-254.613821398558
10	11	0.000100745924	-0.025729114769
10	12	0.006862612483	-1.752327181270
10	13	0.000000001711	-0.000000433362
10	14	0.000000004209	-0.000001079883
10	15	0.000673909075	-0.175740613655
11	1	0.000002901012	-0.000741756108
11	2	0.000002753624	-0.000704069610
11	3	-0.000000082891	0.000021178112
11	4	0.000000026510	-0.000006812176
11	5	0.000000040978	-0.000010478230
11	6	-0.000000060746	0.000015495261
11	7	-0.000000102692	0.000026257292
11	8	0.000000025182	-0.000006405952
11	9	0.006862612483	-1.752327181270
11	10	0.000100745924	-0.025729114769
11	11	1.000000000000	-254.668346901996
11	12	0.000012250633	-0.003133915911
11	13	0.000000001942	-0.000000497447
11	14	0.000000002624	-0.000000671246
11	15	-0.004481866953	1.145988508418
12	1	0.000002753624	-0.000704069610
12	2	0.000002901012	-0.000741756108
12	3	0.000000026510	-0.000006812176
12	4	-0.000000082891	0.000021178112
12	5	-0.000000060746	0.000015495261
12	6	0.000000040978	-0.000010478230
12	7	0.000000025182	-0.000006405952
12	8	-0.000000102692	0.000026257292
12	9	0.000100745924	-0.025729114769
12	10	0.006862612483	-1.752327181270
12	11	0.000012250633	-0.003133915911

12	12	1.000000000000	-254.668346901996
12	13	0.000000002624	-0.000000671246
12	14	0.000000001942	-0.000000497447
12	15	-0.004481866953	1.145988508418
13	1	0.004912623856	-1.253724342774
13	2	0.000000000121	0.000046249978
13	3	-0.003927085588	1.002213251301
13	4	0.000000000009	-0.000036724649
13	5	-0.001524981480	0.388832524387
13	6	0.000000000004	0.000029129707
13	7	0.001215555874	-0.309936829626
13	8	-0.000000000015	-0.000023220796
13	9	0.000000004209	-0.000001079883
13	10	0.000000001711	-0.000000433362
13	11	0.000000001942	-0.000000497447
13	12	0.000000002624	-0.000000671246
13	13	1.000000000000	-254.227996150002
13	14	0.000000000000	0.000002557288
13	15	-0.000000138269	0.000035359765
14	1	0.000000000121	0.000046249978
14	2	0.004912623856	-1.253724342774
14	3	0.000000000009	-0.000036724649
14	4	-0.003927085588	1.002213251301
14	5	0.000000000004	0.000029129707
14	6	-0.001524981480	0.388832524387
14	7	-0.000000000015	-0.000023220796
14	8	0.001215555874	-0.309936829626
14	9	0.000000001711	-0.000000433362
14	10	0.000000004209	-0.000001079883
14	11	0.000000002624	-0.000000671246
14	12	0.000000001942	-0.000000497447
14	13	0.000000000000	0.000002557288
14	14	1.000000000000	-254.227996150002
14	15	-0.000000138269	0.000035359765
15	1	-0.000620546139	0.158157988733
15	2	-0.000620546139	0.158157988733
15	3	-0.000056276500	0.014337024224
15	4	-0.000056276500	0.014337024224
15	5	-0.000002230361	0.000572269127
15	6	-0.000002230361	0.000572269127
15	7	0.000109442366	-0.027869780779
15	8	0.000109442366	-0.027869780779
15	9	0.000673909075	-0.175740613655
15	10	0.000673909075	-0.175740613655
15	11	-0.004481866953	1.145988508418
15	12	-0.004481866953	1.145988508418
15	13	-0.000000138269	0.000035359765
15	14	-0.000000138269	0.000035359765
15	15	1.000000000000	-254.863916032157

M06-2X energies for diabatic states of individual determinants
-255.8451107918
-255.8451107918
-255.8451182729
-255.8451182729
-255.7331953684

-255.7331953684
-255.7332039399
-255.7332039399
-255.6931510436
-255.6931510436
-255.6977591773
-255.6977591773
-255.3345732966
-255.3345732966
-255.9591170867

Sum of M06-2X/cc-pVDZ energies of NH3 and F2. All relative energies are computed based on this value.
-255.9581648282

Transition density functional correlation energies for off-diagonal H matrix elements are determined as described above. The transition density functional energies for the 5 spin-pairing interactions (four local excitations from F2 and one from NH3) are obtained by enforcing energy degeneracy of high and low spin components of the triplet states (see above and the text).

5 0
1 2
-0.00117262
3 4
-0.00115870
5 6
-0.00366553
7 8
-0.00367605
9 10
-0.01026250

Spin-adapted states
7
2 1 2 0
H2L
2 3 4 0
Hm2L
2 5 6 0
Hm3L
2 7 8 0
Hm4L
2 9 10 0
H2L
2 11 12 0
ion1
2 13 14 0
ion2
Block-localized Kohn-Sham ground state
1 15

Diabatic reference state for GSVD projection to yield the GDAC diabatic state energies.
7
1 8 1
1 6 1
1 1 1
1 2 1

1 3 1
1 4 1
1 5 1

Summary of results.

Eigs of adiabatic states determined using Spin-adated configurations
Eigen Values and Eigen Vectors in SA-basis Configurations 8
-255.958164828200
1 2 3 4 5
-255.95933004 -255.81726295 -255.81722912 -255.71059828 -255.71050574 (a.u.)
-0.03 3.83 3.84 6.74 6.74 (eV)
-0.73 88.42 88.44 155.35 155.41 (kcal/mol)
-255.73 30924.39 30931.81 54334.57 54354.89 (cm^{-1})

-0.00001 -0.27363 0.96107 0.04658 0.00127
0.00006 0.96077 0.27350 -0.00136 0.05650
-0.00002 0.00514 -0.01800 0.99989 0.02550
-0.00013 -0.02230 -0.00635 -0.02558 0.99996
0.02069 -0.00000 0.00000 0.00000 0.00003
-0.01907 -0.00000 -0.00000 -0.00001 0.00002
-0.00000 -0.00507 0.00360 -0.00125 0.00089
0.99947 -0.00023 0.00086 0.00007 -0.00002
6 7 8
-255.70013878 -255.68762392 -255.33449105
7.02 7.36 16.97
161.91 169.76 391.36
56630.17 59376.86 136880.57

-0.00000 -0.00000 -0.00967
-0.00000 -0.00000 0.00772
0.00001 -0.00001 0.00271
-0.00003 -0.00002 -0.00210
0.45023 0.89270 -0.00000
0.88977 -0.45612 0.00000
-0.00000 0.00000 1.00000
0.01280 -0.03074 -0.00001

Hdiab in nonorthogonal diabatic basis:
-255.817203
0.027708 -255.710830
8.527190 -0.022016 -255.710846
0.020366 0.000814 -0.039585 -255.959117
-0.019102 7.130609 0.024266 0.224620 -255.817194
0.000014 0.000005 0.000020 1.627396 -0.001452
-255.697773
0.000019 0.000003 -0.000089 -0.249505 0.000424
-1.784914 -255.690357
Sdiab:
1.000000
-0.000108 1.000000
-0.033323 0.000086 1.000000
-0.000080 -0.000003 0.000155 1.000000
0.000075 -0.027866 -0.000095 -0.000878 1.000000

-0.000000 -0.000000 -0.000000 -0.006338 0.000006
1.000000
-0.000000 -0.000000 0.000000 0.000953 -0.000002
0.006961 1.000000
Adiabatic State energies of Non-Orthogonal GSVD
1 2 3 4 5
-255.95933004 -255.81726294 -255.81722912 -255.71059829 -255.71050575

0.00006 0.96078 0.27352 -0.00136 0.05649
-0.00002 0.00515 -0.01800 0.99989 0.02550
-0.00013 -0.02230 -0.00635 -0.02558 0.99996
0.99947 -0.00023 0.00086 0.00007 -0.00002
-0.00001 -0.27369 0.96109 0.04657 0.00128
-0.01907 -0.00000 -0.00000 -0.00001 0.00002
0.02069 -0.00000 0.00000 0.00000 0.00003
structural weights
-0.00000000 0.92379801 0.07489229 0.00000083 0.00130887
0.00000000 0.00006520 0.00080689 0.99847675 0.00065116
-0.00000000 0.00121097 0.00009874 0.00065094 0.99803935
0.99907307 0.00000001 -0.00000001 0.00000000 -0.00000000
0.00000001 0.07492581 0.92420209 0.00087148 0.00000061
0.00048179 0.00000000 0.00000000 0.00000000 0.00000000
0.00044514 0.00000000 0.00000000 0.00000000 0.00000000

==
Gram-Schmidt orthogonalized diabatic states
Hdiab in GS-orthogonal diabatic basis (eV) :
-0.026 3.836 6.730 6.738 3.841 7.088 7.290

-0.026
-0.000 3.836
0.000 -0.000 6.730
0.001 0.067 0.000 6.738
0.000 0.000 0.135 0.000 3.841
0.138 0.000 0.000 -0.000 -0.000 7.088
-0.154 -0.000 0.000 0.000 -0.000 -0.134 7.290

in Hartree:
-255.959117
-0.000009 -255.817203
0.000005 -0.000002 -255.710830
0.000032 0.002474 0.000002 -255.710563
0.000002 0.000009 0.004961 0.000009 -255.817000
0.005056 0.000000 0.000000 -0.000001 -0.000000 -255.697699
-0.005663 -0.000000 0.000000 0.000000 -0.000000 -0.004924 -255.69
0277
S(GS):
1.000000
0.000000 1.000000
0.000000 0.000000 1.000000
-0.000000 0.000000 -0.000000 1.000000
0.000000 -0.000000 0.000000 -0.000000 1.000000
0.000000 0.000000 0.000000 -0.000000 0.000000 1.000000
-0.000000 -0.000000 0.000000 0.000000 -0.000000 0.000000 1.00
0000
adiabatic state energies in GS-orthogonal diabatic (GS-V basis)

1	2	3	4	5	6	7
-0.032	3.834	3.835	6.737	6.739	7.021	7.362
0.99961	-0.00007	-0.00001	0.00002	0.00013	0.00759	-0.02700
0.00006	0.96151	0.27380	-0.00061	0.02317	0.00000	-0.00000
-0.00002	0.01277	-0.04479	0.99859	0.02555	0.00001	-0.00001
-0.00013	-0.02227	-0.00643	-0.02557	0.99940	-0.00003	-0.00002
-0.00001	-0.27358	0.96072	0.04655	0.00128	-0.00000	-0.00000
-0.01893	-0.00000	-0.00000	-0.00001	0.00002	0.89289	-0.44989
0.02069	-0.00000	0.00000	0.00000	0.00003	0.45022	0.89268

GS-V energies and vectors (in input SA basis)

-0.026	3.836	6.730	6.738	3.841	7.088	7.290
0.00000	0.00002	0.00001	-0.00000	1.00035	-0.00000	0.00000
-0.00000	0.99998	0.00010	0.03335	-0.00005	0.00000	-0.00000
-0.00000	-0.00001	1.00000	-0.00008	0.02788	0.00000	0.00000
0.00000	0.00000	0.00000	1.00055	0.00008	-0.00000	-0.00000
0.00000	0.00000	0.00000	-0.00000	-0.00000	0.00000	1.00002
-0.00000	-0.00000	-0.00000	0.00000	0.00000	1.00002	-0.00697
-0.00000	-0.00386	-0.00145	0.00101	0.00479	-0.00000	0.00000
1.00000	0.00008	0.00000	-0.00015	0.00088	0.00634	-0.00100
